# Risk Factors for Anemia in Adolescent Pregnancies: Evidence From the Lembah Pantai Urban Health District in Malaysia

**DOI:** 10.7759/cureus.68094

**Published:** 2024-08-29

**Authors:** Min F Wong, Kavita Jetly, Noriklil Bukhary bt Ismail Bukhary, Vanitha K. Krishnan, Jasmine Avalani Chandrakant, Sin Wan Tham

**Affiliations:** 1 Centre for Health Policy Research, Institute for Health System Research, National Institutes of Health, Ministry of Health, Shah Alam, MYS; 2 Herbal Medicine Research Centre, Institute for Medical Research, National Institutes of Health, Ministry of Health, Shah Alam, MYS; 3 Health Office of Lembah Pantai District, Health Department of Kuala Lumpur and Putrajaya, Ministry of Health, Putrajaya, MYS; 4 Communicable Disease Control, Health Department of Kuala Lumpur and Putrajaya, Ministry of Health, Putrajaya, MYS; 5 Centre for Burden of Disease Research, Institute for Public Health, National Institutes of Health, Ministry of Health, Shah Alam, MYS

**Keywords:** anemia, risk factor, prevalence, urban dwellers, teenage pregnancy

## Abstract

Introduction

Adolescent pregnant mothers are at higher risk of acquiring anemia during pregnancy, which has detrimental consequences for the mother and baby. Untreated and prolonged anemia can lead to perinatal morbidity and mortality and antepartum, intrapartum, and postpartum maternal morbidity. Anemia among adolescent pregnant mothers can be attributed to many factors, such as poor nutrition and the physiological demands of pregnancy. Hence, this study aimed to determine the prevalence and characteristics of anemia among adolescent pregnancies in Lembah Pantai Health District in an urban setting.

Materials and methods

This cross-sectional study utilized secondary data from registry records of adolescent pregnant mothers in the Lembah Pantai District Health Office in Kuala Lumpur, Malaysia from 2019 to 2022. All adolescent pregnancies aged 10 to 19 years were included in this study. Those with a prior diagnosis of thalassemia or other hematological disorders, as indicated in their medical records, were excluded from the study. The chi-square (χ^2^) test was applied to elicit the association at a univariate level, and those variables with a p-value of <0.25 were included in a multiple logistic regression model.

Results

A total of 307 records were reviewed, and 272 pregnant adolescent cases were eligible for the analyses. The prevalence of anemia among teenage pregnancies from 2019 to 2022 was 28.7% (95% CI: 23.0-33.6). This prevalence was lower than in many lower-income countries and developing countries. Factors that were significant on using multiple logistic regression analysis included marital status (unmarried) (AOR: 1.87, 95% CI: 1.01-3.45, p=0.046), residing area Seputeh (AOR: 0.29, 95% CI: 0.14-0.61, p=0.001), and third trimester of first antenatal visit/booking (AOR: 5.08, 95% CI: 2.02-12.70, p<0.001). The population in the Seputeh area has a higher overall household income in comparison to Lembah Pantai.

Conclusions

According to the WHO classification, the prevalence of anemia among adolescent pregnant women at the Lembah Pantai District Health Office reflects a moderate public health issue. Consequently, anemia may exacerbate the impact of other health conditions in this population. Hence, local health authorities should ensure that anemia is adequately addressed without ignoring other crucial health issues. A multidisciplinary, comprehensive, holistic strategy involving healthcare providers, school health teams, and community assets offers a potential solution. Strategies that can be employed include providing targeted health and nutrition education in schools through school health teams. Additionally, establishing adolescent-friendly health clinics tailored to the specific needs of pregnant adolescents and enhancing antenatal services to ensure comprehensive screening and management of anemia should also be implemented. Hence, this will ensure achieving safe motherhood goals through an effective healthcare response.

## Introduction

The World Health Organization (WHO) defines adolescent pregnancy as pregnancy occurring before the age of 20 [1,2]. According to data from the United Nations International Children's Emergency Fund (UNICEF), approximately 15% of women under the age of 18 globally have experienced childbirth [3]. It is worth noting that adolescent mothers account for 11% of all births worldwide [2]. In Malaysia, the annual incidence of underage pregnancies is approximately 14 per 1000 girls [2]. A recent national Malaysian survey conducted in 2022 revealed that the prevalence of current adolescent pregnancy was 2.0% [4]. Adolescent pregnancy is defined as the pregnancy of a woman aged 10 to 19 years of age in Malaysia [5].

It is imperative to recognize that the distribution of adolescent pregnancies varies significantly across different geographical regions, socioeconomic statuses, and cultural and educational backgrounds. Adolescent pregnancy is particularly prevalent in developing countries [3]. Although there has been a positive decline in adolescent pregnancies worldwide over the years due to strong public health initiatives, ongoing and coordinated efforts are necessary to meet the needs of young pregnant mothers and prevent complications during pregnancy and childbirth for both the mother and child [3].

Anemia is widely recognized as one of the most common health issues during pregnancy. The WHO defines anemia as a hemoglobin level below 11 g/dL during pregnancy [6]. Globally, the prevalence of anemia in pregnancy stands at 38%, with over half of Asian women experiencing anemia at some point during their pregnancy [7]. Numerous studies have indicated a higher incidence of anemia among younger mothers [6-8]. In Malaysia, a study conducted in the North-Western Region in 2015 reported a prevalence rate of 53.1% among pregnant adolescents [6]. Undoubtedly, anemia poses significant complications in pregnancy, particularly for adolescent mothers [6].

The heightened risk of anemia among adolescents can be attributed to the physiological demands of pregnancy on a developing adolescent body, further exacerbated by an unfavorable socioeconomic environment [6-9]. These young women, already in a disadvantaged situation, often face early school dropout, unemployment, and a lack of support during pregnancy [1,6]. Due to delayed initiation of antenatal care, their anemic condition is often diagnosed in the later stages of pregnancy [6]. Inadequate antenatal care exposes adolescent mothers to increased risks of pregnancy and birth complications. According to Hayward (2011), adolescent mothers have a 25% higher likelihood of delivering low-birth-weight babies. When coupled with anemia, their risks of premature birth, low birth weight, and even mortality are further amplified [6-8].

The social and health consequences adolescent mothers face are valid public health concerns. Despite overall improvements in living conditions worldwide, access to quality education, healthcare services, employment, and financial security remains challenging for most pregnant adolescents. Being pregnant at a young age impacts the adolescent's family where they reside [2]. In societies where such outcomes are frowned upon, pregnant adolescents often face stigma, which further isolates them from accessing social and healthcare support [10]. A systematic review conducted in Malaysia highlights that the prevalence of anemia in pregnancy was found to be higher in rural compared to urban areas [11]. A few local studies have demonstrated a higher prevalence of anemia in pregnancy in rural areas compared to urban areas. For instance, the prevalence of anemia from a survey done in northwest Malaysia from 2012 to 2015 was 53.1% [6]. Another study from Terengganu, which was conducted in 2009, reported 57.4% [12] and 33.0% in an urban area in Selangor (conducted from 2007 to 2008) [13]. In contrast, the nationwide study by Haniff et al. in 2005 revealed that anemia prevalence is higher in urban areas than in rural areas, with rates of 38% in urban regions and 31% in rural areas [14].

Based on a systematic review of anemia among pregnant mothers in Malaysia by Abd Rahman et al. in 2022, there is a lack of progressive improvement in the prevalence of anemia in pregnancy in this country despite the public health strategies that bring about concern about the effectiveness of the current intervention [11]. Besides that, there is a concerning trend regarding the prevalence of anemia among pregnant mothers in Malaysia.

This study aimed to determine the prevalence and associated factors of anemia in adolescent pregnancy, specifically in an urban setting in Lembah Pantai Health District, from 2019 to 2022. The findings can provide a better understanding of the anemia status among antenatal mothers, thus facilitating healthcare providers to assess, take appropriate action, and manage anemia more effectively. Besides, stakeholders can also benefit from the findings of this study by making informed decisions regarding the burden and implementing targeted programs for those at higher risk of anemia during pregnancy. This aids in resource allocation and has significant cost-effectiveness implications.

## Materials and methods

Study design and location

This cross-sectional study utilized secondary data from registry records of adolescent pregnant mothers registered in the Lembah Pantai District Health Office of Kuala Lumpur, Malaysia from 2019 to 2022. The Lembah Pantai Health District Office, one of the five districts governed by the Kuala Lumpur and Putrajaya Health Department, comprises three sub-districts: Bukit Bintang, Seputeh, and Lembah Pantai. These sub-districts collectively serve an estimated population of 600,000 individuals [15]. This district consists of several government health clinics that provide primary healthcare services to the local population. These services include outpatient care, maternal and child health services, immunizations, family planning, and chronic disease management. The district was chosen for its central location in Kuala Lumpur, a region noted for its high urbanization, dense population, and some of the lowest median income levels in the city.

Study population

This study included all adolescent pregnancies aged 10 to 19 years registered in the Lembah Pantai District Health Office from 2019 to 2022. This registry involves those followed up in the public healthcare clinics under the jurisdiction of Lembah Pantai District Health Office. Those diagnosed with thalassemia or other blood disorders were excluded from the study to ensure the results were not confounded by the physiological or clinical characteristics associated with these conditions. Thalassemia and other blood disorders can significantly impact various health parameters, including hemoglobin levels.

Sampling and sample size calculation

A sample size calculation was performed for each objective of this study, and the largest sample size was used (refer to Table 4 in the Appendix section). A similar study was done by Jusoh et al. (2019), where the prevalence of anemic adolescent mothers was reported to be 53.1%. Using G-power and the OpenEpi 3.01 calculator, the estimated sample size needed was 369. Parameters utilized in this calculation include the proportion and the effect size of the relevant factors like employment status, education level, and booking timing for antenatal care (refer to Table 4 in the Appendix section). However, the total number of adolescent pregnant mothers registered in Lembah Pantai Health District was 307 from 2019 to 2022; hence, this study utilized universal sampling.

Study variables

A comprehensive literature review and discussions with stakeholders, including mother and child healthcare stakeholders, were conducted to confirm the variables of interest to be included in the study. The independent variables used included sociodemographic variables (residing area, age group, ethnicity, marital status, highest education level, employment status, household income) and antenatal variables (parity, trimester of first antenatal visit/booking, fetal weight). The definitions of the variables have been provided in the codebook in the appendices.

The dependent variables included the anemic status of the pregnant adolescent mother (measured by hemoglobin level at booking). A hemoglobin level of less than 11 gm/dl detected at first booking in a health facility was considered anemic. This cut-off aligned with the classification from the WHO and the Malaysian Perinatal Care Manual [16].

Data management and data analysis

Dependent and independent variables were labeled and coded in the Excel sheet before being transferred to the statistical software. Data compilation and analysis were performed using Jamovi 2.3.13. The dataset was first explored for data distribution and missing data. The average missing data across the variables is approximately 5%, except fetal weight, which accounts for 11%. The analysis was conducted both with and without fetal weight, and the outcomes remained unchanged. Given the small dataset, no data points were removed, as evidence suggests that missing data below 5% does not adversely impact the study's conclusions and can be considered negligible [17]. The outcome variable, anemia status, was dichotomized into "0" for no anemia and "1" for the presence of anemia.

In descriptive analysis, categorical variables were described as frequency and percentage. Bivariate chi-square (χ^2^) tests were applied to elicit the association for the final multiple logistic regression analysis. Based on the Hosmer-Lemeshow model development strategy criteria, variables with a p-value of <0.25 were entered into the final model, with the backward exclusion of any variables with a p-value of more than 0.05 [18]. The final model was assessed for multicollinearity and goodness-of-fit (via the Hosmer-Lemeshow test, the area under the receiver operating characteristics curve (ROC), and classification accuracy). Statistical significance was kept at a p-value of 0.05. Multiple logistic regression was performed in this study when a binary outcome was required to define the presence (<11 gm/dl) and absence of anemia (≥11 gm/dl). This statistical method also provides meaningful insights into how different predictors influence the likelihood of the outcome via odds ratio, can handle multiple independent variables, and offers several goodness-of-fit tests and diagnostic measures to determine how well the model fits the data and predicts outcomes.

Ethical consideration

This study obtained ethical approval from the Medical Research and Ethics Committee and was registered under the National Medical Research Register (NMRR), NMRR ID-23-01166-FVD. There were no significant ethical issues since secondary data was used in this study. All anonymized data was kept in an external hard drive for at least seven years and will be destroyed after 10 years. Researchers adhered to the principles of the Declaration of Helsinki and the Malaysian Good Clinical Practice Guidelines.

## Results

Sociodemographic and antenatal

Of 307 adolescent pregnancy cases, 272 cases (a response rate of 88.6%) were eligible for the analyses (fulfills the inclusion criteria). Table 1 shows the sociodemographic and antenatal characteristics of the study sample. The most common age group of adolescent mothers was between 16 and 18 years old (55.9%). Malay accounted for the majority at 192 (70.6%), followed by Chinese at 31 (11.4%), Indians at 28 (10.3%), and others at 8 (2.9%). Half or 149 (55.9%) of the adolescent mothers were single. Most of them received secondary school education (84.6%), and almost all (97.3%) dropped out of school post-delivery. Half had a household income of RM 1000 to RM 1999 150 (55.1%) and the majority were unemployed 213 (78.6%). Regarding antenatal characteristics, most mothers were primigravida 221 (81.3%), with at least three-quarters booked in the second 130 (48.7%) and third trimesters of the pregnancy 69 (25.8%).

**Table 1:** Sociodemographic and antenatal characteristics of adolescent pregnant mothers registered under the Lembah Pantai District Health Office (n=272)

Variables	Variable categories	n	%	Missing data	
				n	%
Sociodemographic characteristics					
Age group	< 16	14	5.1	0	0
	16 to 18	152	55.9		
	> 18	106	39		
Ethnicity	Malay	192	70.6	0	0
	Chinese	31	11.4		
	Indian	28	10.3		
	Sabah and Sarawak	13	4.8		
	Others	8	2.9		
Highest education level	Primary	24	8.8	0	0
	Secondary	230	84.6		
	Tertiary	2	0.7		
	Not schooling	16	5.9		
Employment status	Employed	58	21.4	1	0.4
	Non-employed	213	78.6		
Household income	No income	47	17.3	0	0
	< RM 1000	25	9.2		
	RM 1000-1999	150	55.1		
	RM 2000-2999	41	15.1		
	RM 3000-3999	7	2.6		
	≥ RM 4000	2	0.7		
Marital status	Married	122	45	1	0.4
	Non-married	149	55		
School dropout	Schooling	7	2.7	17	6.3
	Non-schooling	248	97.3		
Residing area	Lembah Pantai	98	38.3	16	5.9
	Seputeh	92	35.9		
	Bukit Bintang	27	10.6		
	Others	39	15.2		
Antenatal characteristics					
Parity	First	221	81.3	0	0
	Second	44	16.2		
	Third	7	2.5		
Trimester of first antenatal visit/booking	First	68	25.5	5	1.8
	Second	130	48.7		
	Third	69	25.8		
Fetal weight at delivery	≤ 2.5 kg	30	12.3	29	10.7
	> 2.5 Kg	213	87.7		
Anemia status	Non-anemic	194	71.3	0	0
	Anemic	78	28.7		

Prevalence and factors associated with anemia among adolescent pregnant mothers

The prevalence of anemia among adolescent pregnancies from 2019 to 2022 was 28.7% (95% CI: 23.0-33.6). Bivariate analysis showed that marital status (p=0.014), trimester of first antenatal visit/booking (p<0.001), and residing areas (p=0.004) were significantly associated with anemia among adolescent pregnant mothers (Table 2). However, only variables with a p-value of <0.25 were entered into multiple logistic regression analysis.

**Table 2:** Association between the characteristics of adolescent pregnant mothers with anemia status (n=272)

Variables	Variable categories	Anemia status				χ^2^ test	p-value
		Anemic		Non-anemic			
		n	%	n	%		
Age group	< 16	7	50.0	7	50.0	3.389	0.184
	16 to 18	43	28.3	109	71.7		
	> 18	28	26.4	78	73.6		
Ethnicity	Malay	59	30.7	133	69.3	1.345	0.246
	Non-Malay	19	23.8	61	76.3		
Highest education level	Secondary	68	29.6	162	70.4	0.576	0.448
	Non-secondary	10	23.8	32	76.2		
Employment status	Employed	20	34.5	38	65.5	1.170	0.279
	Non-employed	58	27.2	155	72.8		
Household income	No income	21	44.7	26	55.3	7.800	0.050
	< RM 1000	8	32.0	17	68.0		
	RM 1000-1999	36	24.0	114	76.0		
	RM 2000-2999	13	26.0	37	74.0		
Marital status	Married	26	21.3	96	78.7	6.042	^*^0.014
	Non-married	52	34.9	97	65.1		
School dropout	Schooling	74	29.8	174	70.2	255^a^	0.434
	Non-schooling	3	42.9	4	57.1		
Residing area	Lembah Pantai	38	38.8	60	61.2	13.48	^*^0.004
	Seputeh	15	16.3	77	83.7		
	Bukit Bintang	11	40.7	16	59.3		
	Others	13	33.3	26	66.7		
Parity	First	69	31.2	152	68.8	272^a^	0.165
	Second	8	18.2	36	81.8		
	Third	1	14.3	6	85.7		
Trimester of first antenatal visit/booking	First	10	14.7	58	85.3	23.85	^**^<0.001
	Second	32	24.6	98	75.4		
	Third	35	50.7	34	49.3		
Fetal weight	≤ 2.5 kg	6	20.0	24	80.0	1.766	0.184
	> 2.5 kg	68	31.9	145	68.1		
*: p<0.05; χ²:chi-square; a: Fisher Exact test (expected count less than 5); **: p<0.001							

Prevalence and factors associated with anemia among adolescent pregnant mothers

In the final multiple logistic regression analysis (Table 3), only three variables remain significant: marital status, residing areas, and trimester of first antenatal visit/booking. Compared to adolescent pregnant women who came for early antenatal booking, those who came in the third trimester had five times the odds of experiencing anemia during pregnancy (AOR: 5.08, 95% CI: 2.02, 12.7, p<0.001). The final model also revealed that mothers who stayed in Seputeh areas (AOR: 0.29, 95% CI: 0.14, 0.61, p=0.001) were less likely to develop anemia than Lembah Pantai. Finally, those mothers who were non-married had two times the odds of developing anemia as compared to the married pregnant mothers (AOR: 1.87. 95% CI: 1.01,3.45, p=0.046). The final model is free of multicollinearity (variance inflation factor (VIF)<5), R^2^ of 0.2093, an area under the curve (AUC) of 0.729, and a classification accuracy of 0.734. The Hosmer Lemeshow test shows a good model fit to the data with p>0.05.

**Table 3:** Multivariable logistic regression of the factors associated with anemia status of adolescent pregnant mothers (n=272)

Factors	Categories	B	Crude OR (95% CI)	p-value	B	Adjusted OR (95% CI)	p-value
Marital status	Married	Ref.	Ref.	-	Ref.	Ref.	-
	Non-married	0.85	2.35 (1.35, 4.07)	*0.002	0.62	1.87 (1.01, 3.45)	^*^0.046
Residing areas	Lembah Pantai	Ref.	Ref.	-	-	Ref.	-
	Seputeh	-1.18	0.31 (0.15, 0.61)	^**^< 0.001	-1.22	0.29 (0.14, 0.61)	^*^0.001
	Bukit Bintang	0.08	1.09 (0.46, 2.59)	0.853	0.10	1.11 (0.42, 2.95)	0.834
	Others	-0.24	0.79 (0.36, 1.72)	0.553	-0.34	0.71 (0.30, 1.70)	0.444
Trimester of first antenatal visit/booking	First	Ref.	Ref.	-	Ref.	Ref	-
	Second	0.64	1.89 (0.87, 4.14)	0.109	0.49	1.64 (0.70, 3.86)	0.257
	Third	1.79	5.98 (2.63, 13.57)	^**^< 0.001	1.62	5.08 (2.02, 12.7)	^**^< 0.001
^*^p<0.05; ^**^p<0.001; Ref.: reference; OR: odds ratio; B: beta; CI: confidence interval; AOR: adjusted odds ratio							

## Discussion

Adolescent girls are in a rapid physical development stage, and the requirement for iron is high. However, adolescent girls' iron intake is usually insufficient due to unbalanced food intake and excessive loss of iron from menstruation. This results in anemia and may be further complicated by socio-environmental factors [6]. This has set a precedent for pregnant adolescent mothers, whereby many adolescents have started their pregnancy with pre-existing anemia.

This study revealed a prevalence of anemia of 28.7% among urban adolescent pregnant mothers attending public healthcare facilities in the Lembah Pantai District Health Office. According to the WHO classification, this prevalence indicates a moderate public health problem [19]. This classification has crucial implications for local health policy and resource allocation. It indicates a critical health issue that requires targeted interventions but may not require the highest level of emergency response. Local health authorities might prioritize anemia prevention and treatment strategies to ensure that anemia is adequately addressed without neglecting other critical health issues. This figure is lower than the prevalence reported in a previous local study conducted by Jusoh et al. (2015), which reported a prevalence of 53.1% more than five years ago. The lower prevalence in this study, compared to the survey conducted by Jusoh et al., can be attributed to the fact that the current sample consisted solely of urban participants. In contrast, the latter study included participants from both urban and rural areas. Increased information availability, literacy, and buying power in metropolitan areas contribute to improved health awareness, healthier nutrition, and better lifestyles [11]. However, it is worth noting that other factors might also account for the differences, such as the year the study was conducted and the geographical settings.

On a global scale, the current anemia rate is lower than that found in low-income countries such as Kenya, where the recorded rate was 61% [20], and in developing countries like Turkey, with a prevalence of 36% [21]. However, the anemia prevalence observed in this study was higher than that reported among adolescents in Ethiopia (23.8%), China (8.5%), Korea (5.3%), and the United States of America (USA) (3.8%). Furthermore, it is expected that the anemia status in our country will be better than that of low-income countries, which often face challenges related to poverty, poor nutritional status, and parasitic infestations [22]. The differences between these countries can be attributed to variations in public health initiatives, socioeconomic status, nutrition programs, healthcare education, awareness, healthcare access, and methodological approaches between studies.

Adolescent pregnant mothers who book their antenatal care in the third trimester demonstrate a higher likelihood of anemia, which is consistent with the findings of the study conducted by Jusoh et al. (2015) and Izuka et al. (2023). However, this finding contrasts with the results of the study by Yilmaz et al. (2018), where the trimester did not affect the anemia status of pregnant mothers [6,21,23]. Late booking for antenatal care deprives these mothers of the opportunity to detect and address the presence of anemia. This leads to a potential increase in the risk of adverse pregnancy outcomes. In addition, this situation may be attributed to a lack of awareness about the available services within the community and a lack of knowledge regarding the importance of early and regular care [24]. This concern extends to the awareness of adolescent girls in secondary schools. Evidence indicates that low anemia awareness in this age group may result from inadequate strategies for disseminating health educational information [25].

Unmarried pregnant mothers demonstrated a higher susceptibility to anemia. The study conducted by Jusoh et al. indicated that marital status had a positive association with the occurrence of anemia, but this effect was observed only in the bivariate analysis. Conversely, a study in Ethiopia revealed contrasting results, showing that married adolescents were 1.53 times more likely to experience anemia than their non-married counterparts [26]. The discrepancy in findings may be attributed to various factors. Married mothers may have better support systems than non-married mothers. This support can improve nutrition, well-being, and healthcare during pregnancy, reducing anemia risk. Besides that, single adolescent mothers may perceive themselves as ineligible for antenatal care or choose not to disclose their pregnancies at an early stage due to cultural stigma and discrimination, especially if the pregnancy is out of wedlock [27]. This contributes to late booking and defaulters, which leads to poorer antenatal care, hence contributing to anemia in pregnancy [24].

In terms of geographical location, findings indicate that adolescent mothers in Seputeh are less prone to developing anemia compared to their counterparts in Lembah Pantai. This disparity may be attributed to the lower socioeconomic status of the population in Lembah Pantai, as indicated by lower overall household income [15]. Consequently, it is crucial to implement targeted interventions and preventive strategies aimed at reducing anemia among adolescent pregnant mothers in the higher-risk Lembah Pantai district, ensuring a cost-effective approach to address this issue.

This study represents one of the initial investigations into the anemia status of urban adolescent pregnant mothers in Malaysia. It offers a preliminary assessment of the factors associated with anemia development in this population. However, it is essential to acknowledge that this study's limitations are focused on only one district, using secondary data analysis and universal sampling. To address this limitation and enhance the generalizability of the results, plans include expanding the study to include a larger sample and using a probability sampling approach from multiple districts under the State Health Department of Kuala Lumpur and Putrajaya. Nevertheless, studying this district is significant since it is densely populated, highly urbanized, and has the lowest median income. The findings can be used to represent other districts with similar characteristics. To mitigate potential researcher biases, the protocol was registered before data analysis, minimizing unplanned analyses to achieve desired outcomes.

Future studies could include additional variables such as dietary habits, compliance with iron supplements among adolescents, and access to healthcare. Expanding these studies to incorporate primary data collection and qualitative interviews will help identify barriers to care. This comprehensive approach will provide a more accurate assessment of the determinants of anemia status in adolescent pregnancies, thereby enabling policymakers to make informed decisions and develop targeted strategies to address this significant public health issue.

## Conclusions

In conclusion, the prevalence of anemia at Lembah Pantai Health Office was 28.7%, indicating a moderate public health issue among urban adolescent pregnant mothers. The factors that significantly affect the occurrence of anemia in pregnancy were unmarried women, living in certain areas, and late booking in the third trimester. Hence, frequent monitoring, follow-up, and targeted health education for those at risk of developing anemia during pregnancy should be conducted. Other appropriate interventions, such as adolescent-friendly health services, further explore its potential to reduce anemia-related antenatal complications to achieve safe motherhood and sustainable developmental goals.

## Appendices

**Table 4:** Sample size calculation

Objectives	References	Estimated sample size with 10% inflation
Prevalence/proportion [5]	Prevalence of anemic teenage mothers: 53.1%	410
Risk factors [5]	-	-
Educational level tertiary secondary and below	OR 2.18	80
Employment status employed unemployed	OR 2.29	90
Adequate antenatal care late booking	OR 16.0	25

**Table 5:** Codebook of the current study DOSM: Department of Statistics Malaysia

Independent variables	Coding	Definition	Scale of measurement
Participants’ ID	ID	This ID is the running number for the dataset	In numerical ascending order
Year	Year	Indicates the respective year of data registry or entry	According to the Gregorian year
Sociodemographic variables			
Residing area	Residing_area	Based on the health office areas of jurisdiction	1=Lembah Pantai; 2=Seputeh; 3=Bukit Bintang; 4=others
Age	Age_Group	As indicated in the respondent’s year of birth in the identification card The categories of the age group were based on literature review	Year of birth - categorical: 1≤16 years old; 2=16 to 18 years old; 3≥8 years old
Ethnicity	Ethnicity	As stated by the respondents	1=Malay; 2=Chinese; 3=Indian; 4=Sabahan or Sarawakian; 5=others
Marital status	I_4_Status_perkahwinan	As verified by the nurse based on the marital certificate provided by the pregnant mothers	1=single; 2=married; 3=widowed; 4=divorced
Highest education level	Highest_Education	The highest education achieved by the sample and noted in the registry	1=primary; 2=secondary; 3=tertiary; 4=not schooling
Employment status	Employment	As stated by the respondents	1=employed; 2=unemployed
Pregnant mother’s household income	Household_Income	The definition of “household” income is given in the DOSM website: “A household consists of related and/or unrelated persons who usually live together and make standard provisions for food and other living essentials”	1=no income; 2≤RM 1000; 3=RM 1000-RM 1999; 4=RM 2000-RM 2999; 5=RM 3000-RM 3999; 6≥RM 4000
Parity	Parity	Based on the definition by Perinatal Care Manual 3rd Edition. Data extracted from the registry. Parity referred to the total number of fetuses delivered by individual mothers	1=first; 2=second; 3=third
Hemoglobin level	HB_booking	Anemia among adolescent pregnancy was defined as hemoglobin concentration less than 11 gm/dl detected at first booking in health facility	1=non-anemic; 2=anemia
Trimester of first antenatal visit	Trimester_Firstvisit	Based on the definition by Perinatal Care Manual 3rd Edition. Data extracted from the registry	1=first; 2=second; 3=third
School dropout	School_dropout	Based on the data given by the registry	1=no; 2=yes
Low birthweight infants	LBW_2500	Low birthweight infants were considered as birthweight of <2500 gm; prematurity was defined as delivery before 37th week of pregnancy (Nagandla and Kumar, 2020)	1≤2500 gm; 2≥2500 gm
